# Piezocatalytic Foam for Highly Efficient Degradation of Aqueous Organics

**DOI:** 10.1002/smsc.202000011

**Published:** 2020-11-12

**Authors:** Jidong Shi, Wei Zeng, Zhaohe Dai, Liu Wang, Qi Wang, Shuping Lin, Ying Xiong, Su Yang, Songmin Shang, Wei Chen, Lingyu Zhao, Xujiao Ding, Xiaoming Tao, Yang Chai

**Affiliations:** ^1^ Research Centre for Smart Wearable Technology Institute of Textiles and Clothing Hong Kong Polytechnic University Hong Kong 999077 China; ^2^ Institute of Chemical Engineering Guangdong Acadamy of Science Guangzhou 510665 China; ^3^ Center for Mechanics of Solids, Structures and Materials Department of Aerospace Engineering and Engineering Mechanics The University of Texas at Austin Austin TX 78712 USA; ^4^ Department of Materials Science & Engineering Centers for Mechanical Engineering Research and Education at MIT and SUSTech Southern University of Science and Technology Shenzhen 518055 China; ^5^ Department of Applied Physics Hong Kong Polytechnic University Hong Kong 999077 China

**Keywords:** free radicals, nanoparticles, organic pollutants, piezocatalysis, porous, water purifications

## Abstract

Piezoelectric catalysis (piezocatalysis) is a physical/chemical process that utilizes piezoelectric potential for accelerating chemical reactions, in which ubiquitous mechanical energies in nature are used for various catalysis applications, e.g., treating organic water pollutants. Despite the high efficiency achieved by piezocatalytic powders, the particles used tend to diffuse in water systems and are hard to be separated, thus causing secondary pollution. Herein, a free‐standing piezocatalytic foam is designed and fabricated, which is composed of BaTiO_3_ nanoparticles embedded in the PVDF scaffold. The as‐prepared PVDF–BaTiO_3_ composite foam demonstrates outstanding piezocatalytic efficiency in removing aqueous organics among state‐of‐the‐art integral piezocatalytic platforms, which lie in the synergy of piezoelectric materials and abundant interconnected pores within the foam. Significantly, PVDF–BaTiO_3_ foam is further applied for purifying natural water samples, by which the permanganate index of the water sample reduces by nearly 30% after 2 h of treatment. In addition, as a monolithic platform, PVDF–BaTiO_3_ foam is easy to be collected, with high reuse stability and applicability for treating various pollutants, resulting in dominant advantages over powder‐based systems for practical high‐flux wastewater treatment. Herein, a piezocatalytic platform is provided for the effective degradation of organic pollutants in water, with minimal environmental side effects.

## Graphical abstract

A piezocatalytic foam composed of PVDF and BaTiO_3_ is prepared with interconnected porosity. The foam demonstrates outstanding efficiency in the piezocatalytic degradation of various organic pollutants, with good reuse stability and minimum environmental side effects. The foam is applied for the purification of natural water samples, by which the permanganate index of the water sample is dramatically reduced.

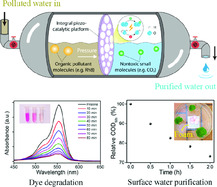

## 1 Introduction

Water pollution has been a great environment concern throughout the world.^[^ ^1^, ^2^ ^]^ Specially, organic pollutants, such as dyes, fertilizers, and food wastes, are massively generated and released into open water from the industry, agricultural activities, and daily life. Scalable and cost‐effective approaches to remove organic pollutants in the aqueous environment have been pursued in recent decades.^[^ ^3^, ^4^, ^5^, ^6^ ^]^ Generally, the main routes for removing organic pollutants are classified into physical separation and chemical degradation. Compared with physically separating the pollutants from the water body, which necessitates a secondary treatment, the chemical approaches can permanently convert the organic pollutants into nontoxic small molecules, such as water, carbon dioxide, and minerals.^[^ ^7^ ^]^ Among various chemical approaches, advanced oxidation processes (AOPs), defined as the oxidation of water pollutants via hydroxyl radicals (⋅OH), have drawn increasing attention in recent years.^[^ ^8^ ^]^ Traditionally, hydroxyl radicals in AOPs could be generated by chemical,^[^ ^9^, ^10^ ^]^ electrochemical,^[^ ^11^, ^12^ ^]^ or photochemical ways.^[^ ^13^, ^14^, ^15^, ^16^, ^17^ ^]^ Recently, piezocatalysis, a new concept for AOPs, has been proposed and well developed.^[^ ^18^, ^19^, ^20^, ^21^ ^]^ Piezoelectric materials, with noncentrosymmetric crystal structures, can generate a net built‐in electric field upon applied strain.^[^ ^22^, ^23^, ^24^, ^25^ ^]^ The built‐in field not only directly outputs power supply, but also promotes some chemical processes, including the catalytic degradation of organic water pollutants.^[^ ^26^, ^27^, ^28^, ^29^, ^30^ ^]^ In natural water, there are various sources of mechanical energy, such as tides, waves, and turbulences by the movement of aquatic animals. These mechanical energies could be potentially transmitted to the submerged piezocatalysts, whereby the organic pollutants could be oxidized without the input of external reagents or power.

The attempts at leveraging piezocatalysis to treat water pollutants started in the early last decade. Li and coworkers applied barium titanate (BaTiO_3_) microdendrite powders for degrading acid orange 7 (AO7) dye in water.^[^ ^31^ ^]^ Since then, dramatic progress in this field has been made in terms of both material and microstructural innovations. On the one hand, materials with improved piezoelectric coefficients have been prepared and applied in piezocatalysis, such as BiFeO_3_,^[^ ^32^, ^33^ ^]^ ZnSnO_3_,^[^ ^34^, ^35^ ^]^ and so on. On the other hand, novel micro/nanostructures have been designed to enhance piezocatalytic performance. For example, nanoparticles with high aspect ratios could exhibit more deformation upon the same environmental perturbation.^[^ ^36^, ^37^ ^]^ In addition, the increase in the surface roughness of piezocatalysts could introduce more stress concentration as well as enlarge the contact area with reactants, which also boosts the piezocatalytic efficiency.^[^ ^38^ ^]^ Representatively, Wu et al. prepared transition metal dichalcogenides (TMDs) nanoflowers which could completely degrade rhodamine B (RhB) in water in 5 min under ultrasonic vibration, which is the highest piezocatalytic efficiency so far.^[^ ^39^, ^40^ ^]^

**Figure 1:**
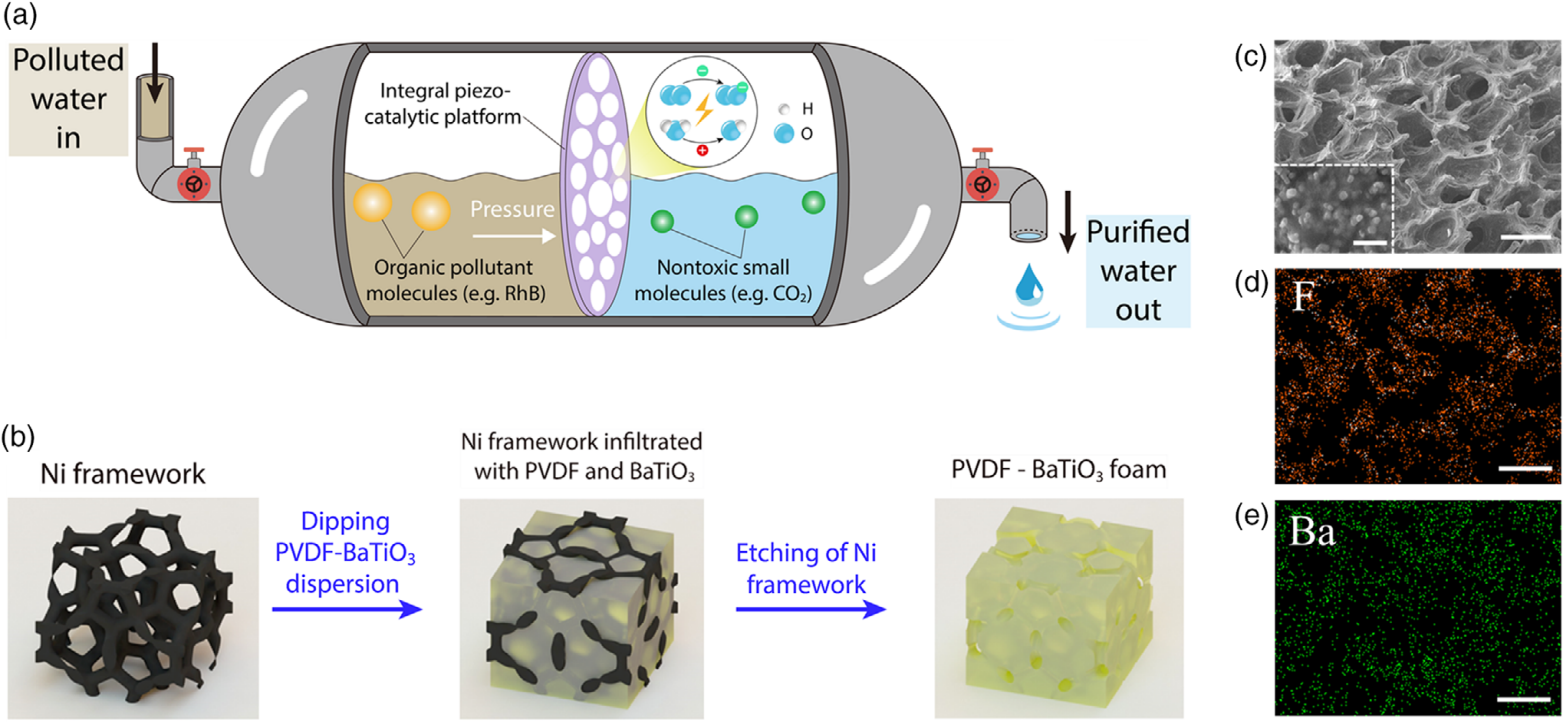
Preparation of PVDF–BaTiO_3_ foam. a) Conceptual illustration of water treatment using an integral piezocatalytic platform. b) Schematic illustration of the PVDF–BaTiO_3_ foam preparation process. c) The SEM image of PVDF–BaTiO_3_ foam in the 30° tilted view. Scale bar: 500 μm. Inset: Magnified view of the PVDF–BaTiO_3_ foam, demonstrating the BaTiO_3_ nanoparticles embedded in the PVDF matrix. Scale bar: 1 μm. d) EDS element mapping of fluorine. e) EDS element mapping of barium. Scale bar for (d) and (e): 500 μm. The corresponding SEM image for (d) and (e) is shown in Figure S5, Supporting Information.

Despite extensive research in applying piezoelectric potential for degrading pollutants (mostly dyes), the high catalytic efficiencies are mostly achieved by dispersing the piezoelectric particles in the solution of target pollutants, which ensures a large contact area for catalytic reactions. However, particulates are difficult to be collected and reused in aqueous solutions. Furthermore, they tend to diffuse and cause secondary pollution in natural water bodies, which greatly restrict their applicability in practical water treatment.^[^ ^41^, ^42^, ^43^, ^44^ ^]^ Therefore, it would be ideal to embed piezoelectric materials in the form of a free‐standing scaffold, where piezocatalytic process could take place under the strike of the water flow (**Figure** **1**a). However, the supporting scaffolds of integral piezocatalytic platforms are usually piezoelectrically inert, which only limits the deformation of piezocatalysts and reduces the accessibility of pollutants to the reaction sites. As a result, the piezocatalytic efficiency of an integral platform is usually inferior to that of piezoelectric nanoparticles.^[^ ^41^, ^42^ ^]^ Acquah and coworkers embedded ZnO nanoparticles in carbon nanotube (CNT) paper for degrading methylene blue (MB) in water, whereas only 12% of the total MB decomposed after ultrasonic treatment for 140 min.^[^ ^43^ ^]^ For the aforementioned TMD nanoflowers with an ultrahigh piezocatalytic efficiency, when embedded into the polydimethylsiloxane (PDMS) substrate, the time for complete RhB degradation increases by more than one order.^[^ ^44^ ^]^ To improve degradation efficiency, integral piezophotocatalytic platforms are developed, which utilize piezoelectric potential for the separation of photogenerated charge carriers.^[^ ^45^, ^46^ ^]^ However, the use of light illumination inevitably increases energy consumption.

To tackle the earlier issues, we developed a piezocatalytic foam with BaTiO_3_ nanoparticles embedded in the porous polyvinylidene fluoride (PVDF) platform. As both PVDF and BaTiO_3_ are piezoelectrically active, the piezoelectric potential generated under stress could be dramatically increased compared with a single piezoelectric component. In addition, the interconnected pores within the PVDF–BaTiO_3_ foam could provide not only a high surface area for piezocatalysis, but also plenty of stress concentration sites, thereby further increasing the piezoelectric potential. As a result, the PVDF–BaTiO_3_ foam demonstrates outstanding piezocatalytic efficiency in treating various organic dyes, with excellent reuse stability. Significantly, the PVDF–BaTiO_3_ foam was applied for the purification of natural surface water samples, and the permanganate index of the water sample decreased dramatically with ultrasonication time. The high piezocatalytic efficiency, together with minimal environmental side effects, endows PVDF–BaTiO_3_ foam great potential in practical natural water treatment.

## 2 Results and Discussion

The piezocatalytic PVDF–BaTiO_3_ foam was prepared by casting a sacrificial nickel (Ni) framework (Figure S1, Supporting Information) with PVDF–BaTiO_3_ dispersion, as shown in Figure 1b. The obtained PVDF–BaTiO_3_ foam has a honeycomb structure from the front view, with many open voids on its surface (Figure 1c). From the magnified view of the foam (Figure 1d, inset), BaTiO_3_ nanoparticles are embedded in the PVDF matrix, showing a well‐hybridized structure. The skeleton of the Ni framework template ensures an interconnected pathway within the resultant PVDF–BaTiO_3_ foam, which could be visualized from the cross‐sectional view of the foam (Figure S2, Supporting Information). The PVDF–BaTiO_3_ foam could be prepared in a large area (Figure S3a, Supporting Information) and is highly flexible (Figure S3b, Supporting Information). The energy‐dispersive X‐ray spectroscopy (EDS) pattern of the foam shows no Ni trace, indicating that the Ni framework template is completely removed (Figure S4, Supporting Information). The preparation of PVDF–BaTiO_3_ foam still results in much of wastewater containing Ni^2+^ ions, which can cause pollution. More ecofriendly templates for generating pores should be considered in the future. From the elemental mapping among the foam (Figure 1d,e), the distribution of BaTiO_3_ (represented by Ba) and PVDF (represented by F) is homogeneous over the sample.

**Figure 2:**
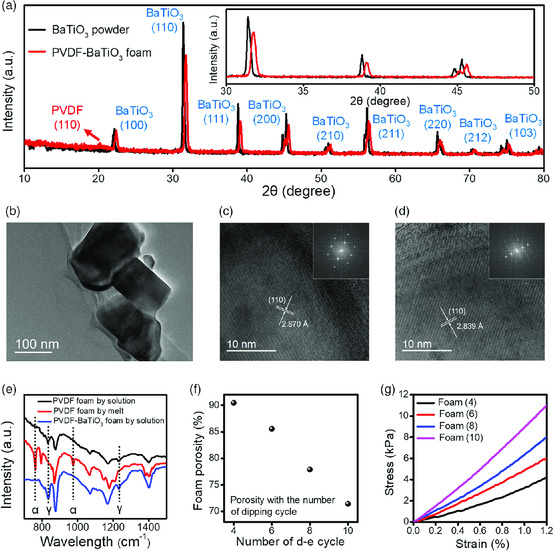
Characterizations of PVDF–BaTiO_3_ foam. a) XRD patterns of BaTiO_3_ powder and PVDF–BaTiO_3_ foam, with characteristic peaks marked. Inset: Magnified view of the patterns, demonstrating the shift of the corresponding diffraction peaks for BaTiO_3_ after composited with PVDF. b) TEM image of BaTiO_3_ nanoparticles. c) HRTEM image of a free‐standing BaTiO_3_ nanoparticle. d) HRTEM image of a BaTiO_3_ nanoparticle in PVDF–BaTiO_3_ composite. The corresponding reciprocal lattice is shown on the upper‐right inset. e) The FTIR spectrum of the solution‐casted PVDF foam, melt‐casted PVDF foam, and solution casted PVDF–BaTiO_3_ foam, with the phase‐indicative peaks marked. f) The porosity of PVDF–BaTiO_3_ foam prepared by four, six, eight, ten D–E cycles. g) The compression stress–strain curve of PVDF–BaTiO_3_ foam prepared by four, six, eight, ten D–E cycles.

To determine the crystalline phase of PVDF and BaTiO_3_, X‐ray diffractometer (XRD) and Fourier transform infrared (FTIR) characterizations were conducted. From the XRD pattern of the foam (**Figure** **2**a), the characteristic peaks for tetragonal BaTiO_3_ and γ‐phase PVDF could be identified. Compared with the XRD pattern of raw BaTiO_3_ powders (Figure 2a, inset), the corresponding diffraction peaks of the foam show a small shift to a higher degree, indicating the existence of compressive stress within BaTiO_3_ after the formation of the composite.^[^ ^47^, ^48^ ^]^ The compressive stress could also be validated by the decrease in interplanar spacing for BaTiO_3_ after forming the composite, as shown in the high‐resolution transmission electron microscope (HRTEM) characterization (Figure 2b–d). The internal stress within the composite guarantees the structural stability of the foam under vigorous ultrasonic agitation in piezocatalysis. From the FTIR spectrum of the foam (Figure 2e), the characteristic peaks for γ‐phase PVDF are clearly identified at 833 and 1234 cm^−1^.^[^ ^49^ ^]^ Notably, the solution‐derived PVDF is also in γ‐phase whereas melt‐derived PVDF is in α‐phase (Figure 2e and Figure S6, Supporting Information), indicating that the final crystalline structure of the as‐prepared PVDF–BaTiO_3_ foam is determined by the crystallization condition, rather than the addition of BaTiO_3_.^[^ ^50^ ^]^ In addition, although the piezoelectric coefficient of γ‐phase PVDF is lower than β‐phase PVDF, it is still one of the largest among polymers.^[^ ^51^ ^]^ As both components in the composite are piezoelectrically active, the application of the foam as a piezocatalyst could be dramatically favored.

As the DMF solvent takes up the majority of PVDF–BaTiO_3_ dispersion, there could be many unfilled spaces after the evaporation of DMF. Therefore, the compactness of the foam could be tuned by repeating the dipping and evaporation (D–E) process for certain cycles. For each D–E cycle, the PVDF–BaTiO_3_ composite could precipitate both on the surface and within the inner pores of the Ni framework after the evaporation of DMF solvent (Figure S6, Supporting Information). After ten D–E cycles, the Ni framework is almost fully covered with PVDF–BaTiO_3_ composites (Figure S7d, Supporting Information). Further increasing the D–E cycles would make it difficult to etch the Ni framework; therefore, the maximum number of D–E cycles in the following discussion is ten. The morphologies of PVDF–BaTiO_3_ foam prepared by four, six, eight, and ten D–E cycles are shown in Figure S8, Supporting Information. The as‐prepared foams become denser as the number of D–E cycles increases, with both the amount and width of the open voids showing a dramatic decrease. In correspondence with morphological evolution, the growing number of D–E cycles also leads to reduced porosity (Figure 2f) and increased modulus of compressibility (Figure 2g). From the mechanical test in Figure 2g, the modulus is about 300 kPa for the foam prepared by four D–E cycles [foam (4)] and about 900 kPa for foam (10). These values are on the same level orders of many silicone rubbers (such as PDMS),^[^ ^52^ ^]^ which imply that the as‐prepared PVDF–BaTiO_3_ foams are quite flexible and deformable.

**Figure 3:**
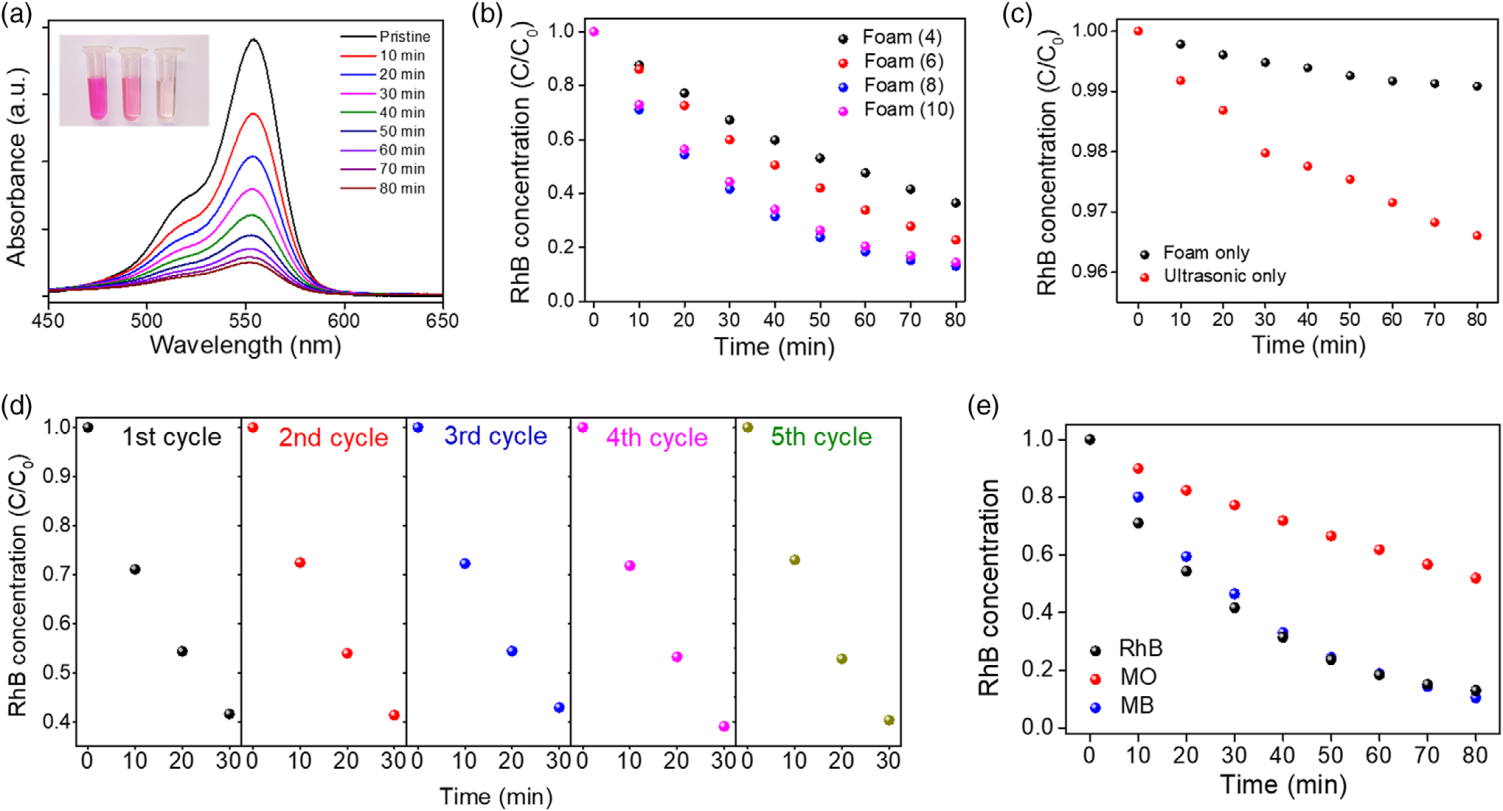
The piezocatalytic performance of PVDF–BaTiO_3_ foam. a) UV–vis absorption spectra of RhB solution in the piezocatalytic process from 0 to 80 min for the foam (8). Inset: The optical image of RhB solution before, 40 min after, and 80 min after the piezocatalytic process, from left to right, respectively. b) The comparison of piezocatalytic efficiency between PVDF–BaTiO_3_ foams prepared by different D–E cycles. c) The degradation of RhB without foam or ultrasonic treatment. d) The piezocatalytic performance for five cycles. e) The comparison of piezocatalytic performance in degrading various dyes.

The piezocatalytic performance of PVDF–BaTiO_3_ foams was then investigated, with organic dye RhB as the target pollutant and ultrasonic treatment as the mechanical input. With the increase in ultrasonication time, the intensity of the absorption peak of the RhB solution shows an obvious decrease in the UV–vis spectrum, indicating the degradation of RhB molecules (**Figure** **3**a and Figure S9, Supporting Information). The piezocatalytic efficiencies of PVDF–BaTiO_3_ foams with different D–E cycles are shown in Figure 3b, which increase from four to eight and then slightly decrease when further increasing to ten. For the highest efficiency obtained by the eight‐times‐dipped sample, 87% of the total RhB is degraded after 80 min of ultrasonication, according to the drop of the absorption peak in the UV–vis spectrum. This efficiency is comparable with most state‐of‐the‐art nanoparticle‐based piezocatalytic systems and superior among the reported integral piezocatalytic platforms (Table S1, Supporting Information). It should be noticed that the degradation of RhB without the existence of foam, or without ultrasonic treatment, is very slow (Figure 3c). The nonpiezoelectric α‐phase PVDF has negligible capability to degrade RhB. (Figure S10, Supporting Information). These control experiments unambiguously suggest that physical adsorption of dye molecules onto the foam and the sonocatalytic process plays a minor role for degrading organics. The outstanding performance can be attributed to not only the synergy of piezoelectric materials, but also the 3D contact between the dye solution and the foam, which is provided by the interconnected pathways left by the etched Ni framework. The nonmonotonic dependence of piezocatalytic efficiency on the number of dipping cycles could be understood as the following trade‐off. The increase in piezocatalytic materials by increasing the number of dipping cycles leads to more electric dipoles under ultrasonication. At the same time, open voids on the foam surface decrease in densities and sizes with the number of dipping cycles, as shown in Figure S8a–d, Supporting Information. For foam (10), the pores on the surface are very few and small (Figure S8d, Supporting Information). As a result, the degraded dyes within the foam cannot be promptly replenished by bulk solution; thus, piezocatalytic efficiency is reduced compared with the eight‐times‐dipped foam.

In addition to the outstanding piezocatalytic efficiency, the PVDF–BaTiO_3_ foam also has a high reuse stability, with a highly repeatable performance among five consecutive cycles (Figure 3d). In addition, the capability of PVDF–BaTiO_3_ foam to degrade other pollutants is also investigated. The evolutions of UV–vis spectra of methyl orange (MO) and MB dyes are shown in Figure S11a and S11b, Supporting Information, respectively. The absorption peak intensity of the two dyes also decreases with ultrasonication time, along with the degradation of RhB. However it is worth noting that the degradation efficiency of MO is much lower than that of RhB and MB (Figure 3e). The reason for the difference can be ascribed to the charge species of their chromophores, which are cations for RhB and MB while are anions for MO.^[^ ^53^, ^54^ ^]^ As both the zeta potentials of PVDF and BaTiO_3_ are negative,^[^ ^55^, ^56^ ^]^ cations tend to be attracted to the foam surface and degraded by the free radicals. By contrast, anions are repulsive to the negatively charged foam surfaces, which is disadvantageous to their piezocatalytic degradation. Furthermore, the piezocatalytic performance of the PVDF–BaTiO_3_ foam under low‐frequency gentle vibration is shown in Figure S12, Supporting Information. Although it is reported that low‐frequency vibration could efficiently separate photoinduced charges, contributing to a good piezo‐photocatalytic performance,^[^ ^57^, ^58^ ^]^ it is insufficient to generate enough piezoelectric charges for high‐efficiency piezocatalysis. Therefore, piezocatalytic efficiency is much lower than the efficiency when using ultrasonication. However, the RhB concentration still decreases by nearly 50% after 8 h, indicating that the foam is still capable for utilizing ambient mechanical energy for piezocatalysis. The reuse stability, applicability for treating various pollutants, and functionality in low‐frequency mechanical input endow the as‐prepared PVDF–BaTiO_3_ foam great potential in practical water‐treatment applications.

**Figure 4:**
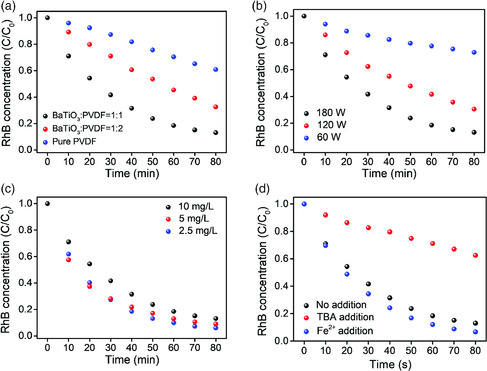
Factors influencing the piezocatalytic performance of PVDF–BaTiO_3_ foam. a) The comparison of the piezocatalytic efficiency in terms of BaTiO_3_ content. b) The comparison of the piezocatalytic efficiency in terms of ultrasonication power. c) The comparison of the piezocatalytic efficiency in terms of the initial RhB concentration. d) The effect of TBA and ferrous ion addition on piezocatalytic efficiency.

We investigate the factors for influencing the piezocatalytic performance. As previously reported, the piezoelectric coefficient (*d*_33_) is ≈−7 pC/N for γ‐phase PVDF^[^ ^51^ ^]^ and 100 pC/N for tetragonal BaTiO_3_.^[^ ^59^, ^60^ ^]^ Therefore, the proportion of BaTiO_3_ in the composite could play a vital role in piezocatalytic performance. Compared with the earlier mentioned 87% RhB degradation after 80 min ultrasonication for the foam with BaTiO_3_:PVDF = 1:1 by mass, the degradation ratio decreased to 67% by reducing the BaTiO_3_:PVDF ratio to 1:2 and to 39%, when there is no BaTiO_3_ in the foam (**Figure** **4**a). These results suggest that both BaTiO_3_ and PVDF in the foam contribute to the piezocatalytic process. On the other hand, although BaTiO_3_ demonstrates a high piezocatalytic efficiency in the nanoparticle form (Figure S13, Supporting Information), BaTiO_3_ foam without PVDF support would break down under ultrasonic treatment. The earlier facts indicate that the PVDF scaffold serves as both the mechanical support and the supplier of piezoelectric potential, demonstrating great superiority as the scaffold for embedding BaTiO_3_ nanoparticles. In addition, the reduction of ultrasonic power also leads to the decrease in piezocatalytic efficiency (Figure 4b), which is easily comprehensible for the less mechanical input. Moreover, we found that piezocatalytic efficiency increases with the reduction of the initial RhB concentration (Figure 4c). For the 2.5 mg L^−1^ sample, the degradation ratio reaches 94% after 80 min. This trend might be related to the reduction of the shielding effect of the adsorbed RhB molecules.^[^ ^31^, ^37^ ^]^ Also, the degradation ratio of RhB is negatively correlated with the volume of RhB solution, for the relatively less amount of piezocatalysts in solution (Figure S14, Supporting Information). Furthermore, to validate the degradation of RhB is a radical‐driven process; two reagents which dramatically affect the behavior of free radicals were added to the piezocatalytic reaction system for exploring their influence on piezocatalytic efficiency. It is reported that *tert*‐butanol (TBA) is a free radical scavenger which consumes the ⋅OH radicals in solution.^[^ ^33^, ^39^, ^42^, ^61^ ^]^ After the addition of TBA, the piezocatalytic efficiency is dramatically reduced (Figure 4d). On the other hand, the ferrous ions (Fe^2+^) can accelerate the generation of ⋅OH through the Fenton reaction,^[^ ^11^, ^62^, ^63^ ^]^ thereby improving the piezocatalytic efficiency in our system (Figure 4d).

**Figure 5:**
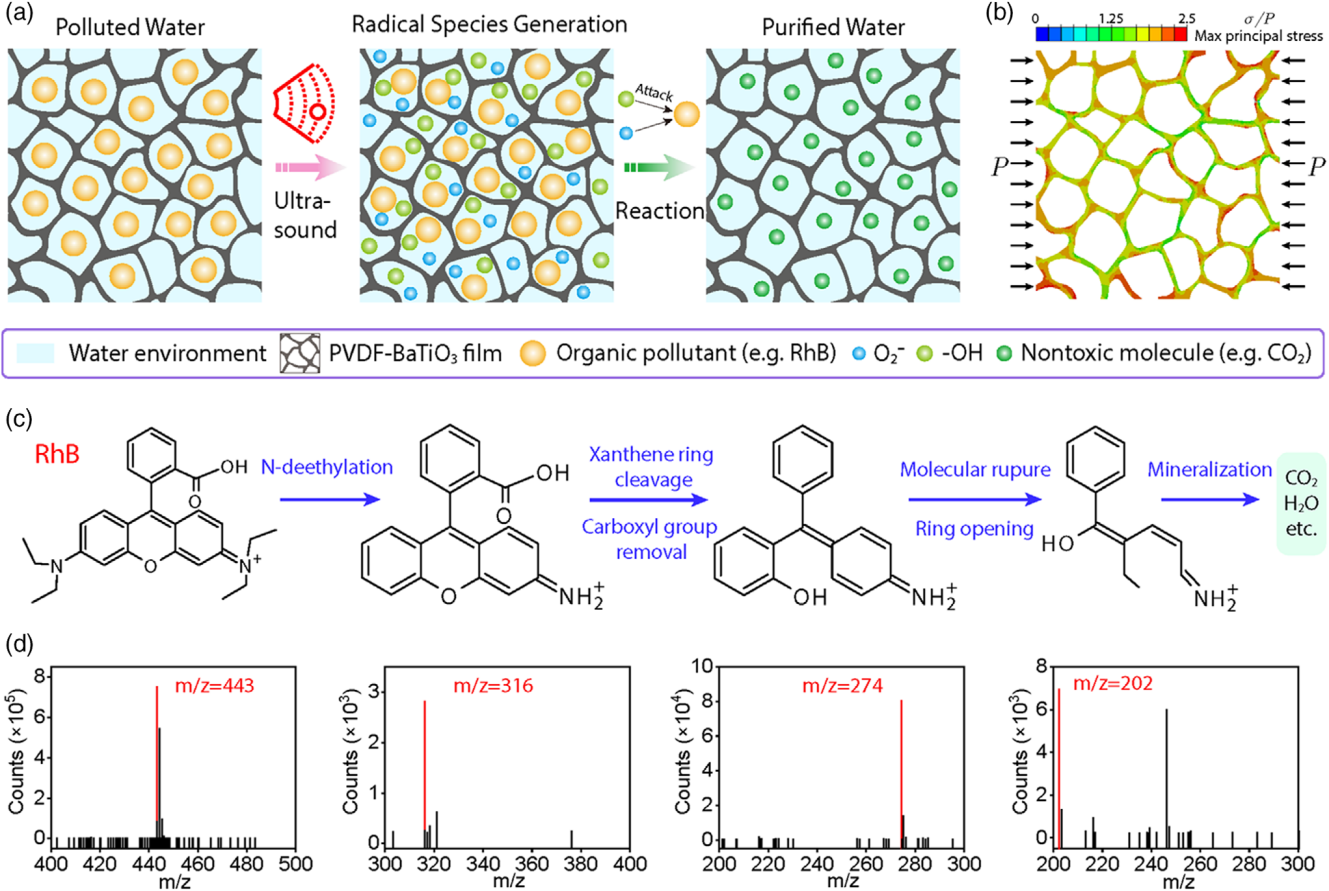
The mechanism of piezocatalysis. a) A schematic illustration of the proposed piezocatalytic degradation process of organic pollutants. b) The stress distribution of a compressed foam using FEA analysis. The stress value is normalized by the applied pressure. c) A brief summarization of the piezocatalytic degradation pathway for RhB, with the detailed summary shown in Figure S15, Supporting Information. d) The corresponding chromatographs for the proposed degradation products in (c). The detailed chromatographs of all products are shown in Figure S16 and S17, Supporting Information.

The piezocatalytic degradation process is schematically shown in **Figure** **5**a. When submerged into the solution of organic pollutants, the interconnected pores within the PVDF–BaTiO_3_ foam could be spontaneously infiltrated by the solution, which provides a high contact area for the reaction. Upon ultrasonic treatment, bubbles emerge in the solution and collapse, which can inflict a high pressure (≈10^8^ Pa) and further generate piezoelectric dipoles (electrons, e^−^, and holes, h^+^) on the foam surface.^[^ ^33^, ^39^, ^42^, ^61^ ^]^ As the energy level of the conduction band is −0.83 V (vs the normal hydrogen electrode, NHE) for BaTiO_3_, the piezoelectric electrons are able to reduce the dissolved O_2_ to ⋅O_2_^−^ (with a standard redox potential of −0.33 V vs NHE). On the other hand, the energy level for the valence band is 2.31 V versus NHE, by which the piezoelectric holes are capable of oxidizing OH^−^ to ⋅OH (with a standard redox potential of 1.9 V vs NHE) in solution.^[^ ^18^, ^61^ ^]^ The resultant free radicals (⋅OH and ⋅O_2_^−^) could attack the organic pollutants and convert them into small and nontoxic molecules through various degradation pathways. The whole process can be written as follows.

(1) $$\text{PVDF}-{\text{BaTiO}}_{3}\text{ foam}+\text{ultrasonic treatment}\to e^{-}+h^{+}$$

(2) $$e^{-}+O_{2}\to \cdot O_{2}^{-}$$

(3) $$h^{+}+{\text{OH}}^{-}\to \cdot \text{OH}$$

(4) $$\text{RhB}+\cdot O_{2}^{-}/\cdot \text{OH}\to \text{degradation products}$$

The porosity of PVDF–BaTiO_3_ foam can not only provide a high surface area for the piezocatalytic process, but also facilitate the concentration of ultrasonic cavitation pressures. To understand the role played by micropores within the foam in piezocatalysis, we conducted finite element analysis (FEA) to simulate the stress distribution in the foam under applied pressure. As shown in Figure 5b, for a foam with patterned holes, most area within the foam is subjected to a stress more than the input pressure, demonstrating a remarkable stress concentration effect. This behavior is particularly beneficial for the generation of large‐amplitude piezoelectric fields and further, a high piezocatalytic efficiency.

To investigate the piezocatalytic degradation pathway of RhB, liquid chromatography (LC)–mass spectroscopy (MS) characterization was conducted to determine the degradation products in this process. The degradation pathway is shown in Figure 5c and Figure S15, Supporting Information, with chromatographical evidence shown in Figure 5d, Figure S16, and Figure S17, Supporting Information. The decomposition of RhB starts with the sequential loss of four ethyl groups, as evidenced by the *m/z* = 415, 387, 359, 331 intermediates.^[^ ^64^, ^65^ ^]^ The loss of the amino group takes place next, evidenced by the *m/z* = 316 intermediate, which ends the *N*‐de‐ethylation process. Notably, as there is no trace of the *m/z* = 334 intermediate (with the ethyl group but without amino group), the loss of ethyl groups must precede the loss of amino groups. The cleavage of xanthene ring (chromophore) follows the *N*‐de‐ethylation process, which also accounts for the color loss of RhB molecules. The coexistence of *m/z* = 333 and 318 intermediates indicates that xanthene cleavage may even occur before the loss of the amino group in the *N*‐de‐ethylation process. The following stages include the removal of the carboxyl group and the opening of the benzene ring, as shown by *m/z* = 290, 274, 246, 218, and 202 intermediates.^[^ ^38^, ^66^ ^]^ These intermediates can be finally mineralized into H_2_O, CO_2_, and other inorganic products. From the quantitative comparison of various intermediates between the samples with different degradation ratios, the partially (Figure S16, Supporting Information) and completely degraded samples (Figure S17, Supporting Information) are richer in the intermediates before and after xanthene ring cleavage, respectively, which further validate the earlier‐mentioned degradation sequence.

**Figure 6:**
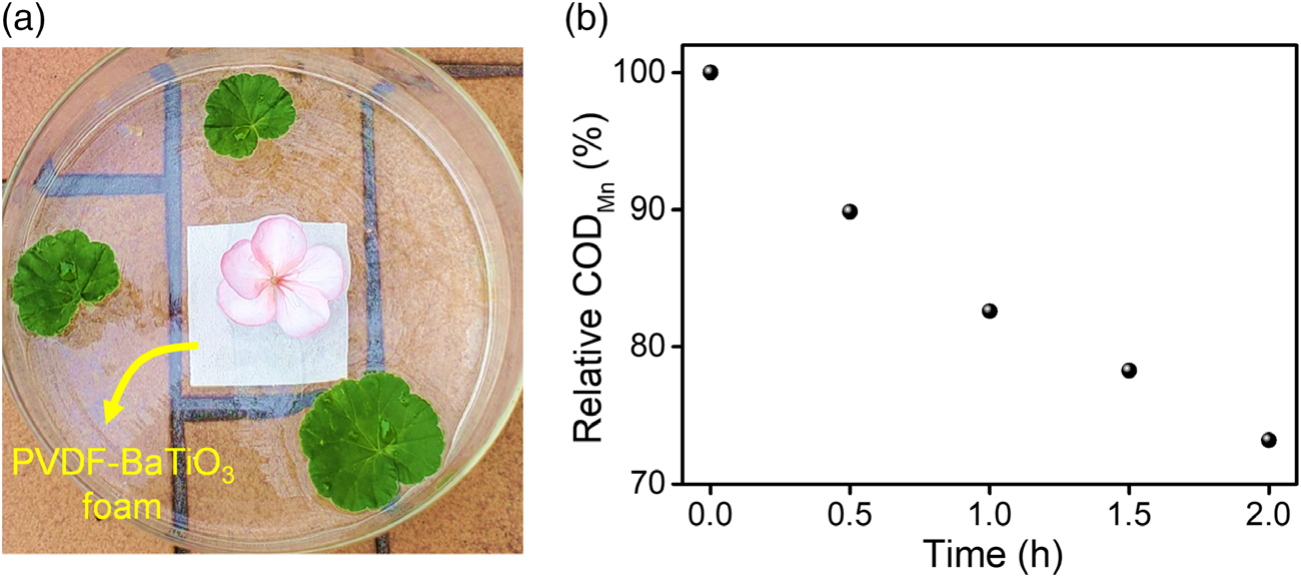
Treatment of natural water samples by PVDF–BaTiO_3_ foam. a) Photo of a simulated piezocatalytic water‐treatment system. b) The evolution of permanganate index with ultrasonication time.

Given the outstanding performance of PVDF–BaTiO_3_ foam for degrading individual dyes, we further attempted to apply the foam for the purification of natural surface water samples (**Figure** **6**a). The content of the organic waste in water resources can be measured by the value of permanganate index, which is a conventional method to quantitatively measure the oxidizable matters in surface water.^[^ ^67^ ^]^ The pristine permanganate index value of the as‐collected water is 2.21 mg L^−1^. We immersed PVDF–BaTiO_3_ foam into the water sample. With the increase in ultrasonication time, the permanganate index value of the water sample shows an obvious decrease and drops nearly 30% after 2 h (Figure 6b). This efficiency is notably inferior to that for RhB degradation measured by absorption peak intensity. From the radical‐triggered degradation pathway of RhB (Figure S15, Supporting Information), there is a big difference between the decolorization (caused by the xanthene ring cleavage) and the complete mineralization of dye molecules. In addition, the organic wastes in the collected natural water samples might be more stubborn, because the easily degradable organics may be consumed by the aqueous organisms. Despite the lower efficiency compared with the mainstream water‐treatment methodologies,^[^ ^7^, ^68^ ^]^ piezocatalytic water treatment based on an integral platform still has advantages such as long‐term environmental friendliness and the potential to be self‐supplied through the natural mechanical input. In practical scenarios, the piezocatalytic foam could be adhered onto a dam, which could perform routine water purification upon the strikes of waves.

## 3 Conclusion

In this study, a piezocatalytic PVDF–BaTiO_3_ foam was developed through a template‐molding process, and it can efficiently degrade the organic pollutants in water. The superior piezocatalytic efficiency of the foam can be ascribed to the synergy of piezocatalytic components as well as the large surface area and stress concentration provided by the internal interconnected pores. In addition, as an integral platform, PVDF–BaTiO_3_ foam is easy to be collected in water, with high reuse stability, which can reduce the side effects on the environment. Significantly, PVDF–BaTiO_3_ foam can be applied for the purification of natural surface water samples, by which the permanganate index decreases dramatically with ultrasonication time. As BaTiO_3_ is also capable for pyro‐ and photocatalysis,^[^ ^69^, ^70^, ^71^, ^72^ ^]^ and PVDF is also a novel platform for loading photocatalysts,^[^ ^73^, ^74^ ^]^ a water‐purification system combining piezo‐, pyro‐, and photocatalysis could also be built based on the PVDF–BaTiO_3_ foam. By introducing temperature fluctuation and light illumination, the efficiency for pollutant degradation could be further improved. With outstanding piezocatalytic performance as well as minimum environmental side effects, the successful development of PVDF–BaTiO_3_ foam not only makes meaningful exploration in the academic research of piezocatalysis, but also provides a valuable solution for water treatment in practical scenarios.

## 4 Experimental Section

4.1

4.1.1

##### The Preparation of PVDF–BaTiO_3_ Foam

PVDF powder (Sigma Aldrich, Molecular weight: 530 000) was dissolved in DMF solution (10 wt%) at 70 °C with mechanical stirring for 5 h. After the solution became clear, BaTiO_3_ nanoparticles (Sinocera Company, 200 nm) were added into the solution, followed by vigorous mechanical stirring. The weight ratio between BaTiO_3_ and PVDF was 1:1, except in the samples used for investigating the influence of BaTiO_3_ content (Figure 3c result). After mechanical stirring for 1 h, the dispersion was dipped onto a porous Ni framework (Lizhiyuan Company, 2 mm thickness, 95–98% porosity). Then, the solution‐infiltrated Ni framework was put into an oven at 60 °C to evaporate the DMF solvent. The dipping process was repeated several times to tune the compactness of the resultant PVDF–BaTiO_3_ foam. After finishing the expected dipping cycles, the PVDF‐ and BaTiO_3_‐infiltrated Ni framework was immersed in 0.5 m ferric chloride (FeCl_3_) solution for etching the Ni template. The PVDF–BaTiO_3_ foam was then rinsed in water and dried in an oven at 60 °C.

##### Characterizations

The morphologies and elemental distribution were characterized by a scanning electron microscope (SEM, TESCAN VEGA3) with an EDS module. The interplanar spacing was conducted by an HRTEM (FEI Tecnai F30). The phase information of the samples was conducted by FTIR spectroscopy (Spectrum 100, Perkinelmer) and an XRD (Rigaku SmartLab 9kW‐Advance). The porosity of the foam was measured by the liquid displacement method.^[^ ^75^, ^76^ ^]^ The LC–MS chromatographs were obtained by combining ultra‐high‐performance LC (UHPLC, Agilent 1290) and MS (Agilent 6540 Q‐TOF).

##### Piezocatalytic Test

A 2.5 cm × 2.5 cm × 2 mm PVDF–BaTiO_3_ foam was immersed in a container bottle filled with 30 mL RhB solution (10 mg L^−1^). The bottle was put on an ultrasonic bench‐top cleaner (Crest Ultrasonics, P1100) for ultrasonic treatment (with effective power of 180 W). The change of RhB concentration was measured by UV–vis absorption spectroscopy (Hitachi, UH5300). The UV–vis absorption spectrum was measured every 10 min, and after each measurement, the solution for the measurement was added again into the bulk solution for further piezocatalysis. To exclude the effect by temperature rise, water in the ultrasonic bath was replaced after each measurement, so the temperature was kept between 25 and 30 °C during ultrasonic treatment. To exclude the photocatalytic effect, the lid of the ultrasonic bath was covered during ultrasonication. For the degradation of MO and MB, the concentration was also 10 mg L^−1^. For verifying the role of free radicals, the concentration of the added TBA and FeSO_4_ was 10 and 5 mg L^−1^, respectively. For the piezocatalytic test under gentle vibration, the bottle was glued on an orbital shaker (A‐Tech global Science Limited), rotating at 240 rpm. For the purification of natural water samples, the water sample was collected in the lily pond of The University of Hong Kong. A 6 cm × 6 cm × 2 mm PVDF–BaTiO_3_ foam was immersed in the 250 mL water sample. The permanganate index of the water sample was measured by a standard processing flow (ISO 8467:1993).

##### Finite Element Analysis (FEA)

The stress concentration of the PVDF–BaTiO_3_ foam was elucidated by the finite element simulation which was conducted by the commercial package Abaqus 2017. For ease of modeling, the foam was modeled as a linear elastic material with Young's modulus of 2 GPa and Poisson's ratio of 0.34. A plane strain condition was adopted. To ensure the linear elastic response, a pressure *p* = 100 kPa was applied to compress the foam, and the distribution of normalized maximum principal stress *σ*/*p* is shown in Figure 4b.

## Conflict of Interest

The authors declare no conflict of interest.

## Supporting information

Supplementary Material

## References

[1] R. P. Schwarzenbach , B. I. Escher , K. Fenner , T. B. Hofstetter , C. A. Johnson , U. Von Gunten , B. Wehrli , Science 2006, 313, 1072. 16931750 10.1126/science.1127291

[2] X. Qu , J. Brame , Q. Li , P. J. Alvarez , Acc. Chem. Res. 2013, 46, 834. 22738389 10.1021/ar300029v

[3] F. Rudroff , M. D. Mihovilovic , H. Gröger , R. Snajdrova , H. Iding , U. T. Bornscheuer , Nat. Catal. 2018, 1, 12.

[4] E. M. Dias , C. Petit , J. Mater. Chem. A 2015, 3, 22484.

[5] J. Wang , Z. Wang , C. L. Vieira , J. M. Wolfson , G. Pingtian , S. Huang , Ultrason. Sonochem. 2019, 55, 273. 30712850 10.1016/j.ultsonch.2019.01.017

[6] M. Panizza , G. Cerisola , Chem. Rev. 2009, 109, 6541. 19658401 10.1021/cr9001319

[7] A. S. Adeleye , J. R. Conway , K. Garner , Y. Huang , Y. Su , A. A. Keller , Chem. Eng. J. 2016, 286, 640.

[8] B. C. Hodges , E. L. Cates , J.-H. Kim , Nat. Nanotechnol. 2018, 13, 642. 30082806 10.1038/s41565-018-0216-x

[9] H. Lee , H.-Y. Yoo , J. Choi , I.-H. Nam , S. Lee , S. Lee , J.-H. Kim , C. Lee , J. Lee , Environ. Sci. Technol. 2014, 48, 8086. 24896837 10.1021/es5002902

[10] X. Duan , H. Sun , Y. Wang , J. Kang , S. Wang , ACS Catal. 2015, 5, 553.

[11] Y. Li , J. Han , B. Xie , Y. Li , S. Zhan , Y. Tian , J. Electroanal. Chem. 2017, 784, 6.

[12] S. Yuan , Y. Fan , Y. Zhang , M. Tong , P. Liao , Environ. Sci. Technol. 2011, 45, 8514. 21866953 10.1021/es2022939

[13] C. Liu , D. Kong , P.-C. Hsu , H. Yuan , H.-W. Lee , Y. Liu , H. Wang , S. Wang , K. Yan , D. Lin , Nat. Nanotechnol. 2016, 11, 1098. 27525474 10.1038/nnano.2016.138

[14] Y. Sang , Z. Zhao , M. Zhao , P. Hao , Y. Leng , H. Liu , Adv. Mater. 2015, 27, 363. 25413166 10.1002/adma.201403264

[15] J. Du , X. Lai , N. Yang , J. Zhai , D. Kisailus , F. Su , D. Wang , L. Jiang , ACS Nano 2011, 5 590. 21189003 10.1021/nn102767d

[16] E. Hu , X. Wu , S. Shang , X. Tao , S. Jiang , L. Gan , J. Clean. Prod. 2016, 112, 4710

[17] E. Hu , S. Shang , X. Tao , S. Jiang , K. Chiu , Polymers 2018, 10, 193. 30966229 10.3390/polym10020193 PMC6415101

[18] Z. Liang , C.-F. Yan , S. Rtimi , J. Bandara , Appl. Catal., B 2019, 241, 256.

[19] M. Wang , B. Wang , F. Huang , Z. Lin , Angew. Chem., Int. Edit. 2019, 58, 7526. 10.1002/anie.201811709 30556295

[20] L. Pan , S. Sun , Y. Chen , P. Wang , J. Wang , X. Zhang , J. J. Zou , Z. L. Wang , Adv. Energy Mater. 2020, 10, 2000214.

[21] Y. Wang , X. Wen , Y. Jia , M. Huang , F. Wang , X. Zhang , Y. Bai , G. Yuan , Y. Wang , Nat. Commun. 2020, 11, 1328. 32165627 10.1038/s41467-020-15015-3 PMC7067860

[22] Z. L. Wang , J. Song , Science 2006, 312, 242. 16614215 10.1126/science.1124005

[23] W. Zeng , X.-M. Tao , S. Chen , S. Shang , H. L. W. Chan , S. H. Choy , Energy Environ. Sci. 2013, 6, 2631.

[24] D. Hu , M. Yao , Y. Fan , C. Ma , M. Fan , M. Liu , Nano Energy 2019, 55, 288.

[25] M. Wang , Y. Zuo , J. Wang , Y. Wang , X. Shen , B. Qiu , L. Cai , F. Zhou , S. P. Lau , Y. Chai , Adv. Energy Mater. 2019, 9, 1901801.

[26] K. Kubota , Y. Pang , A. Miura , H. Ito , Science 2019, 366, 1500. 31857482 10.1126/science.aay8224

[27] H. Mohapatra , M. Kleiman , A. P. Esser-Kahn , Nat. Chem. 2017, 9, 135.

[28] J. Wu , Q. Xu , E. Lin , B. Yuan , N. Qin , S. K. Thatikonda , D. Bao , ACS Appl. Mater. Interfaces 2018, 10, 17842. 29726250 10.1021/acsami.8b01991

[29] J. Feng , J. Sun , X. Liu , J. Zhu , Y. Xiong , S. Tian , Environ. Sci.: Nano 2019, 6, 2241.

[30] S. Lan , J. Feng , Y. Xiong , S. Tian , S. Liu , L. Kong , Environ. Sci. Technol. 2017, 51, 6560. 28447779 10.1021/acs.est.6b06426

[31] K.-S. Hong , H. Xu , H. Konishi , X. Li , J. Phys. Chem. C 2012, 116, 13045.

[32] H. You , Y. Jia , Z. Wu , X. Xu , W. Qian , Y. Xia , M. Ismail , Electrochem. Commun. 2017, 79, 55.

[33] F. Mushtaq , X. Chen , M. Hoop , H. Torlakcik , E. Pellicer , J. Sort , C. Gattinoni , B. J. Nelson , S. Pané , IScience 2018, 4, 236. 30240743 10.1016/j.isci.2018.06.003 PMC6146592

[34] A. Biswas , S. Saha , N. R. Jana , ACS Appl. Nano Mater. 2019, 2, 1120.

[35] M.-K. Lo , S.-Y. Lee , K.-S. Chang , J. Phys. Chem. C 2015, 119, 5218.

[36] C. Jin , D. Liu , J. Hu , Y. Wang , Q. Zhang , L. Lv , F. Zhuge , Nano Energy 2019, 59, 372.

[37] D. Liu , C. Jin , F. Shan , J. He , F. Wang , ACS Appl. Mater. Interfaces 2020, 12, 17443. 32195558 10.1021/acsami.9b23351

[38] J. Wu , N. Qin , E. Lin , B. Yuan , Z. Kang , D. Bao , Nanoscale 2019, 11, 21128. 31682250 10.1039/c9nr07544e

[39] J. M. Wu , W. E. Chang , Y. T. Chang , C. K. Chang , Adv. Mater. 2016, 28, 3718. 26953720 10.1002/adma.201505785

[40] M. Wu , J. Lee , Y. J. Chung , M. Srinivaas , J. Wu , Nano Energy 2017, 40, 369.

[41] W. Qian , K. Zhao , D. Zhang , C. R. Bowen , Y. Wang , Y. Yang , ACS Appl. Mater. Interfaces 2019, 11, 27862. 31305978 10.1021/acsami.9b07857

[42] E. Lin , N. Qin , J. Wu , B. Yuan , Z. Kang , D. Bao , ACS Appl. Mater. Interfaces 2020, 12, 14005. 32142247 10.1021/acsami.0c00962

[43] Y. Gao , S. Li , B. Zhao , Q. Zhai , A. Lita , N. S. Dalal , H. W. Kroto , S. F. Acquah , Carbon 2014, 77, 705.

[44] S. Masimukku , Y.-C. Hu , Z.-H. Lin , S.-W. Chan , T.-M. Chou , J. M. Wu , Nano Energy, 2018, 46, 338.

[45] X. Chen , L. Liu , Y. Feng , L. Wang , Z. Bian , H. Li , Z. L. Wang , Mater. Today 2017, 20, 501.

[46] H. He , Y. Fu , W. Zang , Q. Wang , L. Xing , Y. Zhang , X. Xue , Nano Energy 2017, 31, 37.

[47] N. Ferreira , E. Abramof , E. Corat , V. Trava-Airoldi , Carbon 2003, 41, 1301.

[48] L. Wang , S. Liu , Z. Wang , Y. Zhou , Y. Qin , Z. L. Wang , ACS Nano 2016, 10, 2636. 26745209 10.1021/acsnano.5b07678

[49] X. Cai , T. Lei , D. Sun , L. Lin , RSC Adv. 2017, 7, 15382.

[50] J. Gregorio , M. Cestari , J. Polym. Sci. B Polym. Phys. 1994, 32, 859.

[51] A. Lopes , C. M. Costa , C. Tavares , I. Neves , S. Lanceros-Mendez , J. Phys. Chem. C 2011, 115, 18076.

[52] E. Kroner , R. Maboudian , E. Arzt , Adv. Eng. Mater. 2010, 12, 398.

[53] D. Zhang , J. Li , Q. Wang , Q. Wu , J. Mater. Chem. A 2013, 1, 8622.

[54] Y. Park , Y. Na , D. Pradhan , B.-K. Min , Y. Sohn , CrystEngComm 2014, 16, 3155.

[55] X. Li , Y. Chen , X. Hu , Y. Zhang , L. Hu , J. Membr. Sci. 2014, 471, 118.

[56] Z.-G. Shen , J.-F. Chen , H.-K. Zou , J. Yun , J. Colloid Interface Sci. 2004, 275, 158. 15158393 10.1016/j.jcis.2003.12.025

[57] X. Xue , W. Zang , P. Deng , Q. Wang , L. Xing , Y. Zhang , Z. L. Wang , Nano Energy 2015, 13, 414.

[58] S. Xu , W. Qian , D. Zhang , X. Zhao , X. Zhang , C. Li , C. Bowen , Y. Yang , Nano Energy 2020, 77, 105305.

[59] K.-I. Park , S. Xu , Y. Liu , G.-T. Hwang , S.-J. L. Kang , Z. L. Wang , K. J. Lee , Nano Lett. 2010, 10, 4939. 21050010 10.1021/nl102959k

[60] D. Damjanovic , F. Brem , N. Setter , Appl. Phys. Lett. 2002, 80, 652.

[61] J. Wu , N. Qin , D. Bao , Nano Energy 2018, 45, 44.

[62] W. Lv , L. Kong , S. Lan , J. Feng , Y. Xiong , S. Tian , J. Chem. Technol. Biot. 2017, 92, 152.

[63] J. Peralta-Hernández , Y. Meas-Vong , F. J. Rodríguez , T. W. Chapman , M. I. Maldonado , L. A. Godínez , Water Res. 2006, 40, 1754. 16626778 10.1016/j.watres.2006.03.004

[64] T. Rasheed , M. Bilal , H. Iqbal , S. Shah , H. Hu , X. Zhang , Y. Zhou , Environ. Technol. 2018, 39, 1533. 28513335 10.1080/09593330.2017.1332109

[65] T. Natarajan , M. Thomas , K. Natarajan , H. Bajaj , R. Tayade , Chem. Eng. J. 2011, 169, 126.

[66] C. Lops , A. Ancona , K. Di Cesare , B. Dumontel , N. Garino , G. Canavese , S. Hérnandez , V. Cauda , Appl. Catal., B 2019, 243, 629. 30886458 10.1016/j.apcatb.2018.10.078 PMC6420045

[67] J. Tian , Y. Hu , J. Zhang , J. Environ. Sci. 2008, 20, 252. 10.1016/s1001-0742(08)60039-x 18574969

[68] K. R. Zodrow , Q. Li , R. M. Buono , W. Chen , G. Daigger , L. Dueñas-Osorio , M. Elimelech , X. Huang , G. Jiang , J.-H. Kim , B. E. Logan , D. L. Sedlak , P. Westerhoff , P. J. J. Alvarez , Environ. Sci. Technol. 2017, 51, 10274. 28742338 10.1021/acs.est.7b01679

[69] J. Wu , N. Qin , B. Yuan , E. Lin , D. Bao , ACS Appl. Mater. Interfaces 2018, 10, 37963. 30360057 10.1021/acsami.8b11158

[70] Y. Zhang , P. T. T. Phuong , E. Roake , H. Khanbareh , Y. Wang , S. Dunn , C. Bowen , Joule 2020, 4, 301.

[71] Y. Cui , J. Briscoe , S. Dunn , Chem. Mater. 2013, 25, 4215.

[72] A. A. Yadav , Y. M. Hunge , V. L. Mathe , S. B. Kulkarni , J. Mater. Sci.: Mater. Electron. 2018, 29, 15069.

[73] P. Dong , Z. Huang , X. Nie , X. Cheng , Z. Jin , X. Zhang , Mater. Res. Bull. 2019, 111, 102.

[74] X. Wang , Z. Zhang , Z. Huang , P. Dong , X. Nie , Z. Jin , X. Zhang , Plasmonics 2020, 15, 717.

[75] M. Mulder , Basic Principles of Membrane Technology, Springer Science & Business Media, Dordrecht 2012.

[76] B. Chakrabarty , A. Ghoshal , M. Purkait , J. Membr. Sci. 2008, 309, 209. 10.1016/j.jcis.2008.01.002 18243234

