# Supplementary material for: Piezocatalytic Foam for Highly Efficient Degradation of Aqueous Organics
Source: Small Sci. 2020 Nov 12;1(2):2000011. doi: 10.1002/smsc.202000011 (PMC11935804; doi:10.1002/smsc.202000011)
Supplement: Supplementary file 1 — Supplementary Material [file SMSC-1-2000011-s001.docx]

Copyright WILEY-VCH Verlag GmbH & Co. KGaA, 69469 Weinheim, Germany, 2018.

Supporting Information

Piezo-catalytic foam for highly efficient degradation of aqueous organics

Jidong Shi, Wei Zeng, Zhaohe Dai, Liu Wang, Qi Wang, Shuping Lin, Ying Xiong, Su Yang, Songmin Shang, Wei Chen, Lingyu Zhao, Xujiao Ding, Xiaoming Tao*, Yang Chai*

**Figure S1.**The SEM image of Ni framework template. Scale bar: 500 μm.

**Figure S2.**Cross-sectional view of the PVDF-BaTiO_3_foam.Scale bar: 500 μm.

**Figure S3.** (a) The photo of a 6 cm×6 cm×2 mm PVDF-BaTiO_3_ foam. (b) The photo of a PVDF-BaTiO3 foam bent by fingers.


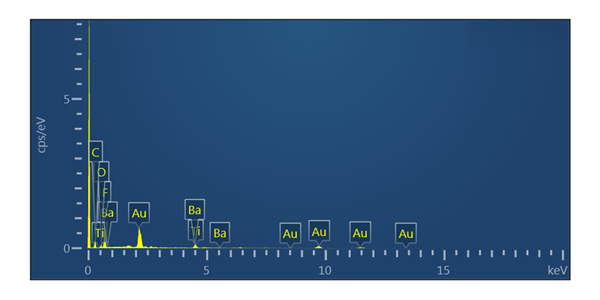


**Figure S4.**EDS pattern of the PVDF-BaTiO_3_ foam, with no trace of Ni element. The existence of Au is due to the sputtering of conductive layer for the preparation of SEM sample.

**Figure S5.** The corresponding SEM image for EDS elemental mapping in Figure 1(d) and Figure 1(e). Scale bar: 500 μm.

**Figure S6.**(a) The XRD patterns of melt- and solution-casted PVDF foam. (b) Magnified view of (a), with indicative marks of certain crystalline planes.

**FigureS7.**The SEM image of PVDF-BaTiO_3_infiltrated Ni framework after 4 (a), 6 (b), 8 (c), and 10 (d) D-E cycles. Scale bar: 500 μm.

**Figure S8.**The SEM image of PVDF-BaTiO_3_ foamafter 4 (a), 6 (b), 8 (c), and 10 (d) D-E cycles. Scale bar: 500 μm.

**Figure S9.**The UV-vis absorption spectrum of RhB solution during 80 minutes piezocatalytic process by PVDF-BaTiO_3_ foam prepared by 4 (a), 6 (b), 8 (c), 10 (d) dipping.

**Figure S10.**The change of RhB concentration with ultrasonication time using α-PVDF powder.

**Figure S11.**The performance of PVDF-BaTiO_3_ for degrading Methyl Orange (MO, a) and Methylene Blue (MB, b).

**Figure S12.** (a) The UV-vis absorption spectrum of RhB solution during the piezocatalytic process using a shaking table. (b) The change of RhB concentration with time.

**Figure S13.** The change of RhB concentration with ultrasonication time using BaTiO_3_ powder.

**Figure S14.**The comparison of RhB degradation ratio for different volume of RhB solution.

**Figure S15.**Schematic illustration of the RhB degradation pathway.

**Figure S16.**The LC-MS chromatographs of various intermediates in partially degraded RhB sample (Degradation ratio:1/3).

**Figure S17.**The LC-MS chromatographs of various intermediates in completely degraded RhB sample.

| **No.** | **Materials&Structures** | **Target pollutant** | **Performance (degradation ratio after certain time)** | **Year** |
| --- | --- | --- | --- | --- |
| 1 | BaTiO_3_ microdendrite powder^[1]^ | AO7 | 60% for 5.7×10^-5^ M, 90% for 2.25×10^-6^ M after 50 min ultrasonication | 2012 |
| 2 | ZnO-Zn nanorod/CNT hybrid film^[2]^ | MB | 80% for 16 mg L^-1^ after 140 min mechanical stirring at 300 rpm | 2013 |
| 3 | ZnO nanoparticles embedded MWCNT paper^[3]^ | MB | 12% for 140 min mechanical stirring at 300 rpm (absence of concentration) | 2014 |
| 4 | MoS_2_ nanoflower^[4]^ | RhB | 98% for 10 ppm after 60 s ultrasonication | 2016 |
| 5 | MoSe_2_ nanoflower^[5]^ | RhB | 90% for 10 ppm after 30 s ultrasonication | 2017 |
| 6 | PZT nanoparticle^[6]^ | RhB | 83% for 10 ppm after 150 min ultrasonication | 2017 |
| 7 | BaTiO_3_ nanowire^[7]^ | MO | 90% for 5 mg L^-1^ after 160 min ultrasonication | 2018 |
| 8 | Annealed BaTiO_3_ nanoparticle^[8]^ | MO | ~92% for 5 mg L^-1^ after 160 min ultrasonication | 2018 |
| 9 | WS_2_ nanoflower embedded in PDMS^[9]^ | RhB | >90% for 10 mg L^-1^ after 90 min ultrasonication | 2018 |
| 10 | Ba_0.8_Sr_0.2_TiO_3_ nanowire^[10]^ | MO | ~100% for 5 mg L^-1^ after 120 min ultrasonication | 2018 |
| 11 | Ag-BaTiO_3_ nanoparticle^[11]^ | MO | 81% for 5mg L^-1^ after 120 min ultrasonication | 2018 |
| 12 | MoS_2_/Pt powders^[12]^ | RhB | ~97% for 10 mg L^-1^ after 40 min ultrasonication | 2019 |
| 13 | BaTiO_3_ embedded in PDMS^[13]^ | RhB | 94% for 5 mg L^-1^ after 120 min ultrasonication | 2019 |
| 14 | ZnO “desert rose” nanoparticle^[14]^ | RhB | ~100% for 2.5 ppm after 180 min ultrasonication | 2019 |
| 15 | Bi_4_Ti_3_O_12_ decussated nanoplates^[15]^ | RhB | ~100% for 5 mg L^-1^ after 80 min ultrasonication | 2019 |
| 16 | ZnSnO_3_ nanoparticle^[16]^ | RhB | ~100% for 4.7×10^6^ M after 120 min ultrasonication | 2019 |
| 17 | Bi_0.5_Na_0.5_TiO_3_-BaTiO_3_ nanofiber^[17]^ | AO7 | ~90% after 60 min ultrasonication (absence of concentration) | 2019 |
| 18 | Ultrathin ZnO/Al_2_O_3_ nanosheet^[18]^ | MO | ~100% for 50 mg L^-1^ after 15 min ultrasonication | 2020 |
| 19 | BaTiO_3_ nanosheets on TiO_2_ nanorod array^[19]^ | MO | ~80% for 5 mg L^-1^ after 60 min ultrasonication | 2020 |
| 20 | MoS_2_-graphene-MoS_2_ sheet^[20]^ | MB | ~100% for 20 ppm after 2 min ultrasonication | 2020 |
| 21 | BaTiO_3_ nanofiber^[21]^ | RhB | 60% for 20 mg L^-1^ after 150 min ultrasonication, 100% for 7.5 mg L^-1^ after 75 min ultrasonication | 2020 |
| 22 | SrTiO_3_ nanoparticle^[22]^ | RhB | 80% for 5 mg L^-1^ after 180 min ultrasonication | 2020 |
| 23 | PVDF-BTO foam (This work) | RhB | 87% for 10 mg L^-1^ and 94% for 2.5 mg L^-1^ after 80 min ultrasonication |  |

**Table. S1.** Comparison of the present work with state-of-art piezo-catalytic systems. Integral systems are marked by blue.

Reference

[1] K.-S. Hong, H. Xu, H. Konishi, X. Li, *J. Phys. Chem. C***2012**, *116*, 13045.

[2] S. Li, M. Zhang, Y. Gao, B. Bao, S. Wang, *Nano Energy***2013**, *2*, 1329.

[3] Y. Gao, S. Li, B. Zhao, Q. Zhai, A. Lita, N. S. Dalal, H. W. Kroto, S. F. Acquah, *Carbon***2014**, *77*, 705.

[4] J. M. Wu, W. E. Chang, Y. T. Chang, C. K. Chang, *Adv. Mater.***2016**, *28*, 3718.

[5] M. Wu, J. Lee, Y. J. Chung, M. Srinivaas, J. Wu, *Nano Energy***2017**, *40*, 369.

[6] Y. Feng, L. Ling, Y. Wang, Z. Xu, F. Cao, H. Li, Z. Bian, *Nano Energy***2017**, *40*, 481.

[7] J. Wu, N. Qin, D. Bao, *Nano Energy***2018**, *45*, 44.

[8] J. Wu, Q. Xu, E. Lin, B. Yuan, N. Qin, S. K. Thatikonda, D. Bao, *ACS Appl. Mater. Inter.***2018**, *10*, 17842.

[9] S. Masimukku, Y.-C. Hu, Z.-H. Lin, S.-W. Chan, T.-M. Chou, J. M. Wu, *Nano energy***2018**, *46*, 338.

[10] B. Yuan, J. Wu, N. Qin, E. Lin, D. Bao, *ACS Appl. Nano Mater.***2018**, *1*, 5119.

[11] E. Lin, J. Wu, N. Qin, B. Yuan, D. Bao, *Catal. Sci. Technol.***2018**, *8*, 4788.

[12] S. Li, Z. Zhao, D. Yu, J.-Z. Zhao, Y. Su, Y. Liu, Y. Lin, W. Liu, H. Xu, Z. Zhang, *Nano Energy***2019**, *66*, 104083.

[13] W. Qian, K. Zhao, D. Zhang, C. R. Bowen, Y. Wang, Y. Yang, *ACS Appl. Mater. Inter.***2019**, *11*, 27862.

[14] C. Lops, A. Ancona, K. Di Cesare, B. Dumontel, N. Garino, G. Canavese, S. Hérnandez, V. Cauda, *Appl. Catal. B-Environ.***2019**, *243*, 629.

[15] J. Wu, N. Qin, E. Lin, B. Yuan, Z. Kang, D. Bao, *Nanoscale***2019**, *11*, 21128.

[16] A. Biswas, S. Saha, N. R. Jana, *ACS Appl. Nano Mater.***2019**, *2*, 1120.

[17] D. Liu, Y. Song, Z. Xin, G. Liu, C. Jin, F. Shan, *Nano Energy***2019**, *65*, 104024.

[18] Q. Nie, Y. Xie, J. Ma, J. Wang, G. Zhang, *J. Clean. Prod.***2020**, *242*, 118532.

[19] E. Lin, N. Qin, J. Wu, B. Yuan, Z. Kang, D. Bao, *ACS Appl. Mater. Inter.***2020**, *12*, 14005.

[20] M. Pan, S. Liu, J. W. Chew, *Nano Energy***2020**, *68*, 104366.

[21] D. liu, C. Jin, F. Shan, J. He, F. Wang, *ACS Appl. Mater. Inter.***2020**, *12*, 17443.

[22] J. Ling, K. Wang, Z. Wang, H. Huang, G. Zhang, *Ultrason. Sonochem.***2020**, *61*, 104819.
